# The Bone Marrow Microenvironment Mechanisms in Acute Myeloid Leukemia

**DOI:** 10.3389/fcell.2021.764698

**Published:** 2021-11-19

**Authors:** Débora Bifano Pimenta, Vanessa Araujo Varela, Tarcila Santos Datoguia, Victória Bulcão Caraciolo, Gabriel Herculano Lopes, Welbert Oliveira Pereira

**Affiliations:** Faculdade Israelita de Ciências da Saúde Albert Einstein, Hospital Israelita Albert Einstein, São Paulo, Brazil

**Keywords:** acute myeloid leukemia, bone marrow, endosteal niche, vascular niche, reticular niche, molecular targets, hematopoiesis, treatment

## Abstract

Bone marrow (BM) is a highly complex tissue that provides important regulatory signals to orchestrate hematopoiesis. Resident and transient cells occupy and interact with some well characterized niches to produce molecular and cellular mechanisms that interfere with differentiation, migration, survival, and proliferation in this microenvironment. The acute myeloid leukemia (AML), the most common and severe hematological neoplasm in adults, arises and develop in the BM. The osteoblastic, vascular, and reticular niches provide surface co-receptors, soluble factors, cytokines, and chemokines that mediate important functions on hematopoietic cells and leukemic blasts. There are some evidences of how AML modify the architecture and function of these three BM niches, but it has been still unclear how essential those modifications are to maintain AML development. Basic studies and clinical trials have been suggesting that disturbing specific cells and molecules into the BM niches might be able to impair leukemia competencies. Either through niche-specific molecule inhibition alone or in combination with more traditional drugs, the bone marrow microenvironment is currently considered the potential target for new strategies to treat AML patients. This review describes the cellular and molecular constitution of the BM niches under healthy and AML conditions, presenting this anatomical compartment by a new perspective: as a prospective target for current and next generation therapies.

## Introduction

The bone marrow (BM) is a soft and viscous tissue within the bone cavities that holds a highly complex and dynamic microenvironment. The BM microenvironment is formed by heterogeneous cells populations, blood vessels and a variety of molecules allocated in niches that provide important regulatory signals to support hematopoiesis, which importantly contribute to the physiological homeostasis throughout life in several aspects, including blood regeneration and immune system maintenance (Yin and Li 2006; Medyouf 2017; Shafat, Gnaneswaran, et al., 2017; Méndez-Ferrer et al., 2020).

The hematopoietic stem cells (HSC) are self-renewing progenitors of the hematopoietic system that reside and remain in the BM until maturation. HSCs and some of their derived subpopulations are dynamically exposed to several stimuli that orchestrate survival, self-renewal, quiescence, migration, and differentiation driving to an adequate hematopoiesis. Considering the hematopoietic cells, there are two core cell lineages in the BM: the common myeloid progenitor line dedicated to megakaryocyte/erythrocyte lineage and to granulocyte/macrophage lineage; and the common lymphoid progenitor line that ultimately originates the lymphocytes (B and T), NK and NKT cell (Passegué et al., 2003; Medyouf 2017; Shafat, Gnaneswaran, et al., 2017).

Homeostasis is affected on many levels by the correct hematopoietic system regulation. For example, immune system cells are associated with neurological functions, endocrine and cardiovascular regulation, metabolism control and cancer surveillance (Malcangio 2019; Ahmari, Hayward, and Zubcevic 2020; Vidal and Pacheco 2020; Klein 2021; Kologrivova et al., 2021). Thus, disturbances in the bone marrow microenvironment may contribute to the development and exacerbation of a range of diseases.

Genes and pathways related to normal development of HSCs may be affected by mutations and trigger hematological oncogenesis with reported changes in the BM microenvironment organization. However, the interaction of leukemia stem cells (LSC) and BM niches are still incompletely described (Passegué et al., 2003; Huntly and Gilliland 2005; Ugel et al., 2015; Méndez-Ferrer et al., 2020). What comes first? BM niches disturbance or LSC arising? How leukemic BM niches might contribute to AML? Such answers may lead researchers toward a next generation of diagnosis markers and therapeutic approaches reaching promising clinical outcomes.

## Acute Myeloid Leukemia: General Aspects and Clinical Features

Acute myeloid leukemia (AML) is a clonal malignant hematopoietic disorder originating from genetic and molecular changes in normal hematopoietic stem cells. As a result, there is a production of immature cells that proliferate and accumulate in the bone marrow (named blasts) (Deschler and Lübbert 2006). These are non-functional cells that compete with and replace normal hematopoietic precursors, which classically leads to cytopenias and leukocytosis (Estey and Döhner 2006). Recent epidemiological data revealed an incidence of 4.3 cases per 100,000 with median age at diagnosis of 68 years and 24% of 5 years survival in United States (Shallis et al., 2019).

Similarly to most of the tumors, AML emerges from accumulation of somatic drivers and secondary mutations (Ley et al., 2013), and diagnosis is currently based on cytogenetic analysis and next generation sequencing (NGS) (Patel et al., 2012). Mutated genes related to signaling pathways and protein kinase activation, such as *FLT3*, are associated to aberrant activation and proliferation of leukemic blasts (DiNardo and Cortes 2016). *FLT3* has a special role in leukemogenesis by collaborating with other mutations, especially those involving the *NPM1* gene. Patients with *NPM1* mutation are stratified as favorable prognostic. However the risk stratification is changed and the clinical course is associated with early relapses when both mutations coexist (*FLT3* and *NPM1*) (Martelli et al., 2013; Papaemmanuil et al., 2016). Other important group of founder mutation are related to genes that regulate epigenetic DNA methylation and chromatin modification. *DNMT3A* mutation, for example, impairs and blocks HSC differentiation and *TET2* mutation impairs myeloid differentiation (Papaemmanuil et al., 2016). Major cytogenetic alterations are also described as responsible for AML development (such as chromosomal translocation, gene amplification, insertion or deletion) and these marks are also important for risk classification and treatment strategy (Short, Rytting, and Cortes 2018; Eisfeld et al., 2020).

AML presents as a heterogeneous disease that typically implicates bone marrow and peripheral blood (PB), and in several cases extramedullary tissues (Narayanan and Weinberg 2020). Pancytopenia (decrease in all blood cell lineages, i.e., anemia, neutropenia, and thrombocytopenia) arises from BM failure and occupation by leukemic blasts.

The common clinical presentation comprises weakness, fatigue, recurrent infections and bleeding disorders (bruise, ecchymosis, epistaxis) (Weinberg et al., 2019). In the leukemic process, fever occurs through 2 mechanisms: neoplastic fever, arising from clonal proliferation process, or due to neutropenia and subsequent recurrent infections. Extramedullary symptoms comprise hepatosplenomegaly, lymphadenopathy, and bone lesions (Rose-Inman and Kuehl 2014).

Coagulation disorders (thrombotic or hemorrhagic) are probably the most severe presentations of AML and can lead patients to death in about 7% of cases (Franchini et al., 2013). All types of AML can present with coagulation impairment although it commonly occurs in acute promyelocytic leukemia (Naymagon and Mascarenhas 2020). Leukostasis is another clinical presentation defined as symptomatic hyperleukocytosis, which is a medical emergency observed in 10–20% of AML patients. Although leukostasis can manifest itself through pathological changes in many organs, the main clinical and potentially fatal symptoms are related to the central nervous system (CNS) and lungs (Stahl et al., 2020).

Diagnosis is set with the presence of 20% blasts in BM or PB, except in a few cases where cytogenetic by itself is sufficient to confirm AML (Arber et al., 2016). Bone marrow aspiration is assessed for several diagnostic tools: morphological analysis, flow cytometry (to define subtypes of the disease), cytogenetic (to search for chromosomal alteration), and NGS (for gene mutations) (Jongen-Lavrencic et al., 2018).

The success of AML treatment is to bring the disease to remission (undetectable blats and molecular marks). There is a well established protocol divided into two stages basically: induction and consolidation therapy (De Kouchkovsky and Abdul-Hay 2016). Intensive induction chemotherapy comprises the first-line treatment and aims at maximum reduction of the leukemic blast count (<5% in BM). For patients eligible for intensive chemotherapy, standard chemotherapy is a combination of Cytarabine for 7 days and Daunorubicin or Idarubicin for 3 days (7 + 3 protocol), and this strategy is responsible for disease remission in more than 50% of patients of all ages since 1970 (Lichtman 2013; Döhner et al., 2017). An initial evaluation is required to determine treatment modalities, for example, patients with advanced age are not candidates for intensive induction chemotherapy. Alternative options can also reflect good results in terms of overall survival and quality of life with hypomethylation-inducers combined with Venetoclax or Cytarabine low dose as recently published (DiNardo et al., 2020; Wei et al., 2020). Consolidation therapy is the second step after induction treatment and aims to improve remission achieved with initial treatment. Depending on risk stratification (Datoguia et al., 2018), the patient can be treated with high-dose chemotherapy or undergo to bone marrow transplant with curative intent (Shimomura et al., 2021).

After a period of remission, relapse episodes are frequent with more drug-resistant leukemic clones leading patients to death due to disease complications, and only 24% of patients achieve a 5-year overall survival (Hirabayashi et al., 2021). Unfortunately, combination of re-induction protocols with target therapies (for example, tyrosine kinase inhibitors) did not produce any longer survival in the patients (Daver et al., 2020).

A specific and distinct subtype of AML classified by the World Health Organization (WHO) and currently referred as APL with *PML-RARA* (O'Donnell et al., 2017) is the Ate promyelocytic leukemia (APL), also named as AML-M3 by the French-American-British (FAB) classification system (Warrell et al., 1993; Puccetti and Ruthardt 2004). It is a separate entity comparing to the others AML by presenting histological and clinical characteristics such as the morphology of Auer’s rods and the propensity to exhibit disseminated intravascular coagulation (Mistry et al., 2003). From a therapeutic point of view, there is also divergence. While AMLs as a whole are treated with 7 + 3 protocol (cytarabine with daunorubicin/or idarubicin), patients diagnosed with low-risk APL receive induction treatment with arsenic trioxide plus all-trans retinoic acid (Burnett et al., 2015), which leads to disease remission with excellent results in terms of overall survival (Wang and Chen 2008). Thus, non-APL AMLs are the main current issue for the scientists.

In recent years, there has been a significant improvement in different features of AML’s physiology with a focus on BM niches, and other drugs have been addressed to interfere with niche-specific cell populations, extracellular matrix components, varied growth factors, and cell adhesion molecules produced by niche cells. Researchers intend to impair leukemia development or progression by interfering in the bone marrow microenvironment, and ultimately improving the clinical outcomes (Zhang et al., 2019; Ladikou et al., 2020).

## Healthy and Leukemic Bone Marrow Niches

Raymond Schofield was the first one to propose the niche concept for the human hematopoietic system. The niches were described as areas in which the hematopoietic stem cells not only reside but may establish associations with other cells to modulate their behavior (Schofield 1978). Currently, niches are commonly defined as microenvironments that combine non-hematopoietic cells and the architecture of the bone marrow to promote self-renewal and differentiation of HSCs by providing invaluable and essential factors (Morrison and Spradling 2008; Szade et al., 2018).

Collectively, the components of the niches orchestrate the phenomenon of hematopoiesis. Significant progress in bone marrow imaging technologies has provided a better understanding of the molecular and cellular complexity of the bone marrow. However, compartmentalization of this space remains a challenge due to the anatomical and functional connectivity and the numerous and simultaneous interactions between the HSCs and the surrounding cells. Nevertheless, many publications divide the bone marrow, geographically, into vascular, endosteal, and reticular niches (Behrmann, Wellbrock, and Fiedler 2018). Although there are no anatomical boundaries to physically segregate the most diverse constituents of the marrow microenvironment, the niches have specific components that define their role on the homeostasis of hematopoietic stem cells.

In summary, the endosteal niche, more hypoxic, keeps the HSCs in a quiescent state, leading to a long-term storage of the hematopoietic cells and regulating the size of the stem and progenitor cells pool in the bone. Oppositely, the vascular niche, more vascularized and rich in oxygen, supports the progenitors that are actively proliferating and differentiating to form the various hematopoietic cell lineages. The reticular niche takes part in the regulation of stem cell factors from surrounding cells secretion. Additionally, there are other cell groups whose addressing within the marrow microenvironment still causes disagreement among scientists, but whose role in the regulation of hematopoietic cells is worthy of being analyzed in this review. Considering how complex the crosstalk among niches and HSC, it is expected how a disturbance of this regulatory and integrating network can lead to, or at least be related to hematological diseases such as leukemias (Ghobrial et al., 2018). Literature provides pieces of evidence about normal and leukemic bone marrow functional architecture, and the next paragraphs will elucidate how AML modifies and takes advantage from this altered BM microenvironment.

### The Physiological Endosteal Niche

The endosteal niche, also entitled osteoblastic niche, is characterized by its proximity to the trabecular or cortical bone (Nilsson, Johnston, and Coverdale 2001; Behrmann, Wellbrock, and Fiedler 2018). It is well established that this compartment is filled with mesenchymal stromal cells (MSCs), osteoprogenitor cells, pre-osteoblasts, mature osteoblasts, osteocytes, and osteoclasts (Le, Andreeff, and Battula 2018).

The mesenchymal stromal cells, also called mesenchymal stem cells or bone marrow stromal cells, are multipotent stem cells capable of renewing themselves and have the ability to differentiate and to give rise to cells such as marrow adipose tissue, bone cartilage and occasionally myofibers. In the endosteal niche, these cells are surrounding pre-osteoblasts and osteoblasts of the bone-lining cell. However, MSCs have also been described in the perivascular region, around sinusoidal endothelial cells (Yusop et al., 2018). MSCs express key hematopoietic factors such as stem cell factor (SCF) and stromal cell–derived factor 1 (SDF-1). They are also sources of trophic factors modulating the immune system and inducing intrinsic stem cells to repair damaged tissues (Prockop 1997, 1998; Bianco et al., 2013; Shafat et al., 2017; Yusop et al., 2018; Waclawiczek et al., 2020; Rodríguez-Fuentes et al., 2021).

The osteoblastic population derived from mesenchymal precursors constitutes the main cell group resident in the endosteum, playing key roles in bone development including the synthesis and mineralization of the extracellular bone matrix (Sugiyama and Nagasawa 2012). In addition, osteoblasts are related to self-renewal and maintenance of HSCs in an undifferentiated and quiescent stage (Le, Andreeff, and Battula 2018).

The cross-talk between osteoblastic cells and HSCs is mediated by several pairs of molecules such as soluble cytokines, cytokine receptors and adhesion molecules (Goulard, Dosquet, and Bonnet 2018). The duo Notch 1/Jagged 1 constitutes an important axis in the regulation of hematopoiesis. Several studies have demonstrated that activation of Notch 1 maintains the immature profile of hematopoietic precursors, both *in vitro* and *in vivo*. The physiological activation of this signaling occurs through the interaction between the transmembrane glycoprotein receptor Notch, present on the membrane surface of hematopoietic precursor cells, and one of its possible ligands such as Jagged 1, a transmembrane protein expressed notably by osteoblasts. The cellular response to this pathway comprehends an increased self-renewal and concentration of the pool of HSCs (Stier et al., 2002).

The noncanonical Wnt signaling axis represents another central pathway associated with the long-term maintenance of quiescent HSCs. Flamingo (Fmi) and Frizzled 8 (Fz8) compose a group of molecules that mediate the activation of this signaling. Fmi is a cadherin family molecule, a mediator of homophilic adhesive interaction between pre-osteoblasts and HSCs. Fz8, in turn, is a protein of seven transmembrane domains coupled to the Fmi that regulate intracellular calcium levels. Together, Fmi and Fz8 impair the nuclear translocation of NFAT, a transcription factor, preventing the expression of IFNγ, a significant molecule in the activation of HSCs (Kimura et al., 2006). Conversely, the canonical Wnt signaling (β-catenin-mediated) is associated with maturation of progenitors under bone marrow stress conditions, meanwhile the role of the canonical route on the maintenance of HSCs is still uncertain. In homeostatic conditions, pre-osteoblasts express noncanonical Wnt ligands and inhibitors of the canonical Wnt pathway. In consequence, the interaction between pre-osteoblasts and HSCs triggers noncanonical Wnt signaling and contribute to HSC quiescence by downregulation of IFNγ production and by antagonization of the canonical Wnt route (Sugimura et al., 2012).

Arai et al. (2004) revealed Tie2/Angiopoietin-1 signaling as another important pathway implicated in the quiescence of HSCs. Tie2 is a tyrosine-kinase receptor expressed in the HSC population, more precisely the long-term repopulating HSCs (LTR-HSCs). In contrast, angiopoietin-1 is a glycoprotein secreted by osteoblasts. Apparently, Ang-1 binding is succeeded by phosphorylation of Tie2, resulting in activation of the phosphatidylinositol 3-kinase (PI3-K)/Akt signaling pathway, which promotes the maintenance of the self-renewal capacity and protection against bone marrow stresses. Furthermore, the Tie2/Ang-1 axis has been shown to be critical for positive regulation of β1-integrin, which allows the maintenance of HSCs in a primitive phenotypic state by adhering to stromal cells (Arai et al., 2004).

Osteoblasts are known to secrete various proteins for the composition of the extracellular matrix (ECM), including collagen (COL) and fibronectin (FN), the main components of ECM of BM microenvironment (Hackney et al., 2002). Ang-1 promotes FN- and COL-mediated cell adhesion of Tie2^+^ HSCs to ECM, and increased levels of Ang-1 stimulate an elevation in the adhesion of HSCs to the bone surface *in vivo*, which may be related to cell survival (Arai et al., 2004).

Upregulation of β1-integrin is also involved in the modulation of HSCs quiescence via the THPO/MPL (thrombopoietin/thrombopoietin receptor) pathway. MPL is the product of the transcription and translation of the c-MPL gene (Vigon et al., 1992). THPO, in turn, was identified as a primary cytokine, a regulator of megakaryocyte development and platelet production (Kaushansky 1995). However, recent studies have demonstrated the role of these molecules associated with self-renewal and quiescence of HSCs in adult BM. Evidence suggests that MPL^+^ HSCs adhere to THPO-producing osteoblastic cells and THPO- or MPL-knockout mice show a decrease in the number of HSCs (Kimura et al., 1998). Experiments involving a neutralizing anti-MPL provoked a reduction in the proportion of quiescent HSCs and HSCs-niche interactions. Furthermore, an increase in the number of HSCs in G0 state was observed by exogenous THPO infusion *in vivo*. Altogether, these observations suggest that THPO/MPL signaling regulates quiescent HSCs and HSC-niche interactions on the endosteal surface and contribute to the hematopoiesis process (Yoshihara et al., 2007).

A study by Susan K. Nilsson et al. provided significant evidence that osteopontin (Opn) is an essential component in the regulation of HSCs. Osteopontin is a phosphorylated glycoprotein that has multiple domains (Chen et al., 1993). The binding of this molecule to its distinct receptors explains the most diverse cellular functions that osteopontin may modulate (Denhardt and Guo 1993). In the context of hematopoiesis, this protein is expressed at high levels by endosteal osteoblasts. Similar to the Tie2/Ang-1 and THPO/MPL axes, Opn-HSCs interaction occurs via β1-integrin. Experiments involving Opn-knockout mice reported a significant increase in HSCs cycling, suggesting that, under normal conditions of osteopontin expression, this molecule acts as a negative regulator of stem cell pool size, actively maintaining the quiescence of these cells by inhibiting the entry of HSCs into the cell cycle and, consequently, blocking cell proliferation (Nilsson et al., 2005; Stier et al., 2005).

A conflicting signaling mechanism is observed on the N-cadherin pathway. N-cadherin is characterized as a cell-cell adhesion molecule dependent on the presence of calcium íons, enabling homophilic interactions between neighboring cells (Song et al., 2002). Studies are suggesting that N-cadherin acts in the regulation of HSCs quiescence. According to the observations of these groups, the HSCs N-cadherin is anchored to the N-cadherin expressed in osteoblasts, and silencing of N-cadherin results in loss of HSCs long-term engraftment (Hosokawa et al., 2010). On the other hand, another evidence indicates that N-cadherin-mediated HSCs-osteoblasts interactions are dispensable for the maintenance of hematopoietic cells in the endosteal surface (Kiel et al., 2009; Greenbaum et al., 2012). Experiments with deletion of encoding N-cadherin from HSCs revealed no change in the behavior of stem cells (Kiel, Radice, and Morrison 2007). Similar results were obtained with the deletion of N-cadherin expressed by osteolineage cells, revealing that the loss of interaction through this pathway did not have a significant effect on the status of the HSCs cycle. Consistent with these studies, it has been increasingly proposed that redundant pathways involving other cadherins such as E-cadherin, C-cadherin, and R-cadherin may compensate the loss of N-cadherin, enabling the maintenance of HSCs quiescent phenotype in the endosteum (Greenbaum et al., 2012).

Osteoclasts are also important cells on the endosteal surface. They are involved in the reabsorption of the mineralized bone matrix, but their role in the regulation of HSCs is controversial. There is evidence that osteoclasts, through secretion of proteolytic enzymes such as cathepsin K, promote the degradation of the endosteal niche components like stromal cell-derived factor 1 (SDF-1), stem cell factor (SCF), and osteopontin, leading to the mobilization of hematopoietic progenitor cells (Kollet et al., 2006). In contrast, one study elucidate the role of osteoclasts in the mobilization of hematopoietic progenitors by ablation of osteoclasts under administration of zoledronate in mice. Results revealed higher mobilization of hematopoietic cells confirming that osteoclasts are not required for the mobilization of HSC-derived progenitors (Winkler et al., 2010). But it is still clear that further studies are needed to clarify in detail the role of osteoclasts in hematopoiesis.

### The Leukemic Endosteal Niche in Acute Myeloid Leukemia

**FIGURE 1:**
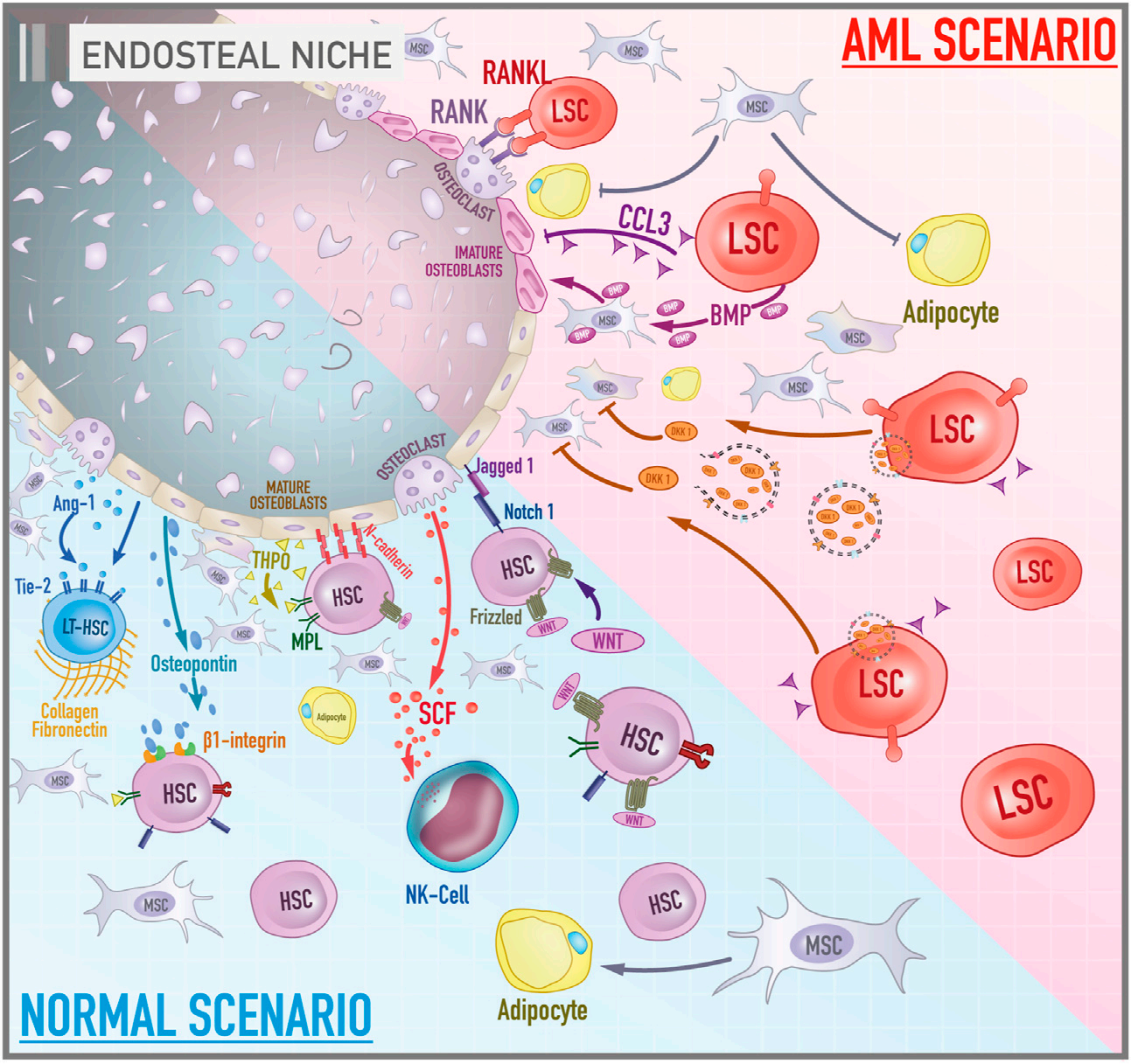
Cellular and molecular components of bone marrow endosteal niche in both healthy and leukemic scenarios. The illustration contrasts the activities and components of the endosteal niche under physiological conditions and during the course of acute myeloid leukemia. In a normal scenario, signaling pathways such as MPL/THPO, Jagged 1/Notch 1, and Ang-1/Tie-2 promote the maintenance of hematopoietic cells in a primitive profile and their capacity for self-renewal. Osteopontin, via β1-integrin, acts as a negative regulator of stem cell pool size by blocking cell proliferation. N-cadherin constitutes a cell adhesion mechanism between HSCs and osteoblasts. In contrast, the role of the canonical Wnt signaling on the maintenance of HSCs is still debated. Collagen and fibronectin constitute the main components of the extracellular matrix of the bone marrow microenvironment. Mesenchymal stromal cells give rise to osteoblasts and adipocytes. In the AML scenario, leukemic stem cells secrete a variety of molecules such as BMP, DKK1, CCL3 that, together, lead to inhibition of adipogenic differentiation of MSCs, promotion of the osteogenic lineage, impairment of osteoblast functioning and an environment rich in immature osteoblasts. In addition, the RANK/RANKL signaling pathway leads to osteoclastogenesis and increased osteoclast survival.

The leukemic endosteal niche is marked by the loss of fine balance between bone formation and resorption, which may be associated with oncogenic events (Figure 1). A critical pathway in bone remodeling is RANK/RANKL. The receptor activator of nuclear factor kappa-B ligand (RANKL) is a membrane protein found on the surface of stromal and osteoblast cells, but which is also expressed by blasts of AML patients. The receptor activator of nuclear factor kappa-B (RANK) is a transmembrane protein found on the surface of osteoclasts. Activation of this axis leads to osteoclastogenesis and increases the survival of osteoclasts (Schmiedel, Grosse-Hovest, and Salih 2013).

Battula et al. revealed that AML cells support leukemogenesis through switching from adipogenic to osteogenesis differentiation of MSCs by a bone morphogenetic protein (BMP)-dependent mechanism. Leukemic cells release BMP that activates Smad 1/5 signaling on MSCs to drive osteoblast lineage differentiation (Battula et al., 2017). Several studies have also reported that the leukemic endosteal niche is marked by osteoprogenitor cells and immature osteoblasts, delaying the maturation process of these cells and promoting deficient bone mineralization. Early-stage osteoblasts markers such as osterix, RUNX2, and Col1a1 are expressed in mesenchymal cells of AML patients. On the other hand, markers of mature osteoblasts such as osteocalcin are not found in AML-MSCs (Battula et al., 2017; Le et al., 2018). Frisch et al. brought a possible explanation for this phenomenon. The chemokine CCL3, also known as macrophage inflammatory protein 1α (MIP1α), secreted by AML cells appears to inhibit osteoblast function decreasing osteocalcin levels in the blood in murine AML model of AML patients (Frisch et al., 2012).

In fact, blocking the terminal differentiation of MSCs into mature osteoblasts seems to contribute to AML progression. Another interesting mechanism is the secretion of DKK1-containing exosomes by AML blast cells (DKK is a negative regulator of osteogenesis and normal hematopoiesis). And the pharmacological inhibition of DKK1 in AML murine model increased mice survival by impairing the progression of the disease. The inhibition of exosome release targeting Rab27a is also able to damage AML progression *in vivo* (Kumar et al., 2018). Together, these studies show how intricate and diverse the apparatus of AML is to manage the endosteal niche to create a proficuous environment for malignant cells.

In addition to the loss of fine control of endosteal physiology, various studies indicated that MSC can interfere with hematologic malignancies via inhibiting the proliferation of tumor cells. The most commonly accepted mechanism is that MSCs induce tumor cell cycle arrest (Fathi et al., 2019; Fathi et al., 2019). Li et al. (2018) showed that Umbilical Cord-MSCs (UC-MSCs) inhibited the proliferation of HL-60 and THP-1 (AML cell lines) by a mechanism dependent on cytokines release (Li et al., 2018; Fathi et al., 2019). Furthermore, *in vitro* studies have shown that bone marrow MSCs can support leukemia progenitor cell survival and provide resistance to cytotoxic therapies (Azadniv et al., 2020). It is reasonable to hypothesize that the MSC population could be associated with minimal residual disease maintenance with low rate of proliferation but high resistance to chemotherapy-induced apoptosis, and AML clone evolution would have important advantages to avoid MSC differentiation. More studies with stratification of sample patients (for instance undifferentiated AML, promyelocytic AML) are necessary to understand not only the clinical and prognostic relevance of the leukemia-endosteal niche relationship, but also to offer new perspectives of coadjuvant treatments and molecular markers.

### The Physiological Vascular Niche

Whereas the osteoblastic niche offers a microenvironment for the maintenance of non-differentiated conditions of stem cells, the vascular niche, according to the literature, acts on their maturation. First of all, the architecture of the vascular niche provides a microenvironment with a higher concentration of oxygen and endocrine growth factors, which, in comparison to the endosteal, stimulates a distinct cellular behaviour. As a result, HSCs assume a proliferative and differentiating profile, which allows the proper generation and release of the hematopoietic populations to the peripheral circulation (Kopp et al., 2005).

**FIGURE 2:**
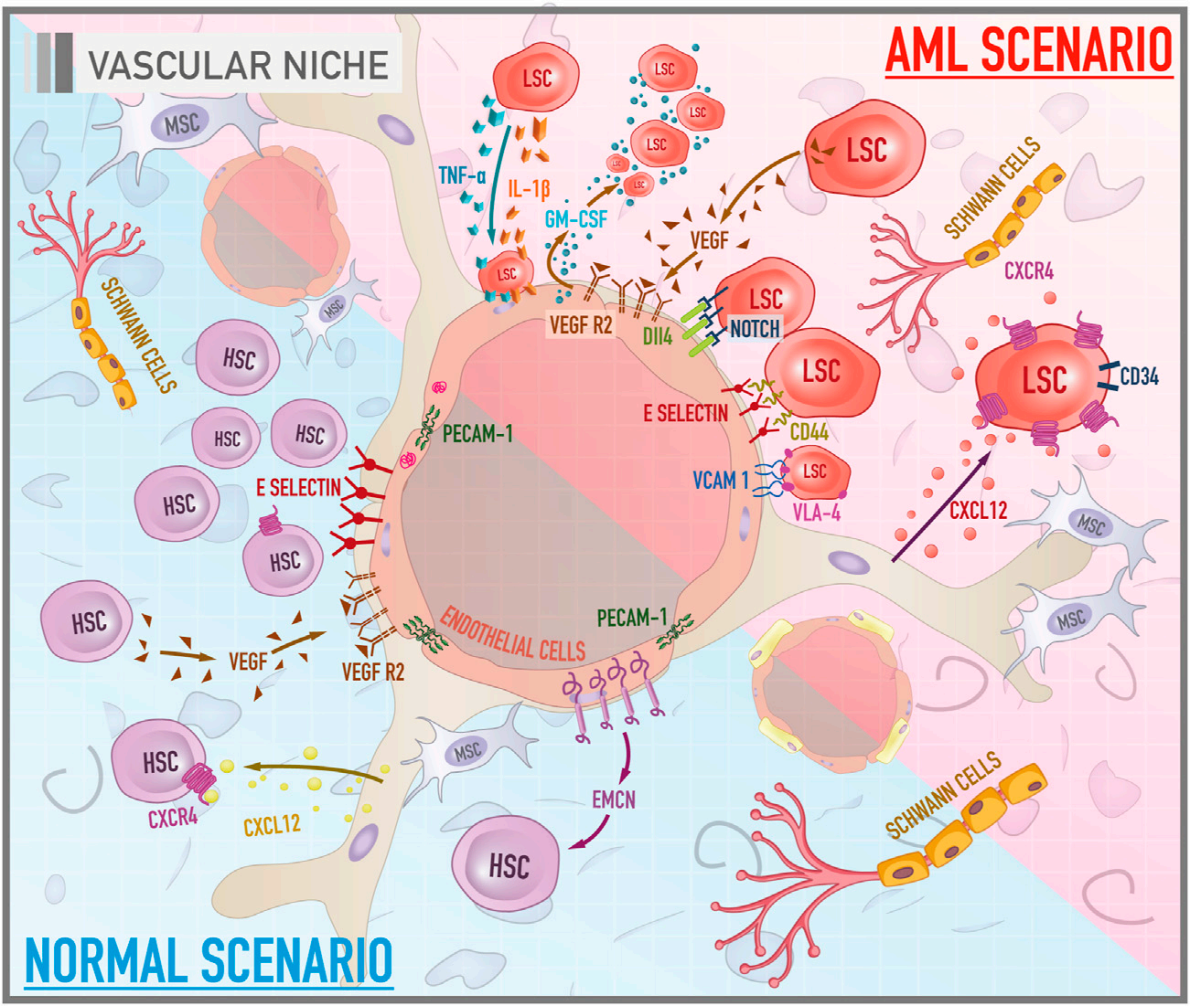
Cellular and molecular components of bone marrow vascular niche in both healthy and leukemic scenarios. The illustration compares the functioning of the vascular niche under a normal context and during leukemogenesis. In a physiological scenario, HSCs acquire a proliferative profile and differentiated phenotype. PECAM-1 stabilizes the endothelial layer. EMCN, in turn, is a potential molecule related to angiogenesis. Another pathway associated with vessel morphogenesis is the VEGF/VEGFR. E-selectin acts as a promoter of HSCs proliferation. In the AML scenario, there are important axes that mediate the anchorage of leukemic stem cells (LSCs) to endothelial cells such as E-selectin/CD44 and VCAM-1/VLA-4. Signaling pathways such as VEGF/VEGFR and Notch/Dll4, in turn, are related to tumor angiogenesis. GM-CSF is a molecule with mitotic properties on LSCs, secreted by VEGF-stimulated endothelial cells. Cytokines, especially IL-1 beta and TNF-alpha, secreted by LSCs promote the attachment of these cells to the endothelium.

The vascular niche (Figure 2), composed primarily of sinusoidal endothelial cells, pericytes, and unmyelinated Schwann cells (glial cells of the peripheral nervous system involving small axons of autonomous post-ganglion neurons), is located in a more centralized area of the bone marrow and can be characterized as an intertwined vessels with vascular arrangements subdivided into sinusoidal and arteriolar endothelium. Stromal cells and extracellular matrix work as surrounding support components (Nombela-Arrieta et al., 2013). This dense vascular network is responsible for the renewal of nutrients, oxygenation of the medullary tissue, and regulation of the entry and exit of cells, such as late plasma cells coming to BM to occupy specific locations and the newly differentiated granulocytes. Blood vessels, in general, are made up of different cell types. The inner layer of the vessel is composed of endothelial cells (ECs), which are covered by perivascular cells called pericytes. These are incorporated into the subendothelial basement membrane and connect the ECs to smooth muscle cells that cover large vessels such as arteries and veins (Sivaraj and Adams 2016). However, bone marrow vascularization, in particular, is mainly formed by a sinusoidal endothelium composed of a single layer of ECs (Tavassoli 1981). This non-stratified endothelial arrangement allows blood cells to pass through a sinusoidal wall easily, supporting transendothelial migration of hematopoietic cells from the medullary tissue into the bloodstream. Thus, the role of the vascular niche includes not only the regulation of HSCs differentiation and proliferation but also the immediate release of the progeny of these cells into the bloodstream (Wright et al., 2001; Kopp et al., 2005).

More specifically, the bone marrow vascularization is configured in a special way: the arteries align longitudinally to the perimeter of the diaphysis of long bones, branching into small arterioles to infiltrate the bone marrow and, finally, forming a network capillary, which is divided into two capillary subtypes, called H and L, which can be distinguished according to their structure, function and surface marker (Kusumbe, Ramasamy, and Adams 2014; Kusumbe et al., 2016; Sivaraj and Adams 2016). In the capillary network, blood flows through type H capillaries, located in the metaphysis, which are connected to the mentioned small arterioles and, later, it flows towards the more permeable type L sinusoidal capillaries, located in the transition between the metaphase and the diaphysis (Kusumbe, Ramasamy, and Adams 2014; Ramasamy et al., 2014). These capillaries can be differentiated by the molecules that are expressed on their surface and by the cells that surround them. Type H capillaries, surrounded by osteoprogenitors, express high levels of CD31 and endomucin. On the other hand, type L capillaries, surrounded by leptin receptor (LEPR)^+^ and CXCL12-abundant reticular (CAR) cells, exhibit low levels of CD31 and endomucin on their surface. Thus, these capillaries form a dense and overly branched sinusoidal network within the medullary cavity, responsible for regulating the HSCs compartment (Sugiyama et al., 2006; Ding et al., 2012). Finally, at the exit of the bone marrow, the capillaries drain into a wide central vein that passes through a massive, calcified bone matrix towards the periphery (Kusumbe et al., 2016).

The deficiency of direct arterial supply contributes to a more hypoxic diaphysis, while the metaphysis, marked by the presence of capillaries, is configured as a better oxygenated region. In consequence, this vascular configuration contributes to the formation of metabolically distinct microenvironments, playing different roles in the regulation of hematopoietic cells. Hypoxia is known to act as an essential regulator of HSCs dormancy (Eliasson and Jönsson 2010; Takubo et al., 2010). Low oxygen levels can affect cellular energy metabolism, redirecting it from oxidative phosphorylation to cytoplasmic glycolysis (Semenza 2007). Hematopoietic cells residing in the endosteal niche have a predominance of glycolysis as energy metabolism, a less efficient process to obtain ATP molecules compared to oxidative phosphorylation prevalent in HSCs residing in the vascular niche. As a consequence, HSCs are induced to assume a dormant state for energy saving (Zhang and Sadek 2014). Another effect of this energy redirection is the lower intracellular production of reactive oxygen species (ROS). The maintenance of HSCs in a quiescent state depends on the careful management of ROS levels. Excessive levels of these substances can lead to damage to the genetic material and its products, which is associated with the development of leukemias, marked by genomic instability (Sallmyr, Fan, and Rassool 2008).

Similar to the endosteal niche, the vascular one also has molecules and signaling pathways that are essential for maintaining the architecture of the microenvironment and regulating HSCs behavior. The platelet endothelial cell adhesion molecule (PECAM-1), for example, also known as cluster of differentiation 31 (CD31) is a transmembrane glycoprotein that forms a significant part of the intercellular junctions of the endothelium and stabilizes the endothelial cell monolayer. This protein belongs to the immunoglobulin family and its properties suggest that it is involved in leukocyte transmigration, angiogenesis, and integrin activation (Hashimoto et al., 2012; Kim et al., 2013; Lertkiatmongkol et al., 2016).

Whether in the organogenesis of bone marrow or in the homeostatic response to several stimuli, angiogenesis is a physiological process co-regulated by osteoblasts and performs an important function in the vascular niche (Schipani et al., 2013).

It is a process that consists of the formation of new vessels that grow from existing vessels through branched morphogenesis, which is a fundamental event for many physiological and pathological processes, such as embryonic development, wound healing, tumor growth, and metastasis (Carmeliet 2005). Several highly orchestrated stages are involved in angiogenesis. First, there is the degradation of the cell matrix by endothelial cells mediated by the release of metalloproteinases, followed by the migration, proliferation, and alignment of these cells. Therefore, the lumen is established and, finally, the anastomosis of the newly formed vessel with adjacent vascular structures occurs (Conway, Collen, and Carmeliet 2001). These phases are regulated by a variety of soluble growth factors as well as cellular interactions.

One of the main regulators of angiogenic responses is the vascular endothelial growth factor (VEGF), a signal protein that acts through the VEGF receptor 2 (VEGFR2) and activates endothelial cells by signaling cascades that allow subsequent branching of the vessel (Leung et al., 1989). The downstream intracellular signaling triggers p42/44 MAPK and PI3K/Akt, which promotes the migration, proliferation, survival and differentiation of ECs (Gerber et al., 1998; Kobayashi et al., 2010). Ang-1, a ligand also derived from pericytes, inhibits the apoptosis of ECs, thereby promoting the regulation of HSCs (Dimmeler and Zeiher 2000). Another molecule with a potential role in angiogenesis is endomucin-1 (EMCN). Experiments involving the modulation of the levels of this glycoprotein, present in the venous and capillary endothelium, showed that EMCN knockdown reduces the migration and proliferation of endothelial cells associated with the suppression of tube formation as well as a reduction in the levels of phospho-VEGFR2, phospho-ERK1/2, and phospho-p38-MAPK, suggesting suppression of the signaling pathway by VEGF. On the other hand, overexpression of EMCN had a positive impact on vessel morphogenesis (Park-Windhol et al., 2017).

The characterization of the vascular microenvironment as a proliferative niche for HSCs is consistent with studies that indicate E-selectin, an adhesion molecule expressed exclusively by endothelial cells, as a promoter of the proliferation of HSCs. Experiments involving administration of an E-selectin antagonist or observation of E-selectin knockout mice reported improvement in the dormant state of HSCs as well as potentiation of the self-renewal capacity, strengthening the idea that E-selectin plays a central role in the proliferation of hematopoietic cells (Winkler et al., 2012).

### The Leukemic Vascular Niche in Acute Myeloid Leukemia

Studies have shown that the vascular microenvironment is consistently altered in the evolution of acute myeloid leukemia (Figure 2). Also, it seems that blast cells have the ability to create conditions that favor their proliferation and survival, which may have important implications for the pathophysiology of leukemia (Wang and Zhong 2018). In fact there is a cross-talk between endothelial cells and leukemic cells through autocrine and paracrine stimuli (Cogle et al., 2014). Stucki et al. demonstrated a synergy of adhesive receptors and cytokines, mainly IL-1 beta and TNF-alpha, secreted by blast cells, to produce the attachment of AML blast cells to the endothelium (Stucki et al., 2001). Important axes that mediate the anchorage of leukemic stem cells (LSCs) to endothelial cells most documented in the literature are CD44/E-selectin and VLA-4/VCAM-1 (Cavenagh et al., 1993). As previously mentioned, E-selectin is an adhesive molecule highly expressed by endothelial cells that can bind to CD44, a glycoprotein widely expressed on the membrane surface of LSCs. The vascular cell adhesion protein 1 (VCAM-1), a member of the immunoglobulin superfamily, is an endothelial ligand for very late antigen-4 (VLA-4), belonging to the β1 subfamily of integrins, expressed by leukemic cells. Together, these adhesive interactions can enable the migration of LSCs through the vascular wall and result in the establishment of disease outside the bone marrow. Some promising studies have revealed that the antagonization of E-selectin and VCAM-1 increases myeloblast mobilization and chemosensitivity, compromising their refuge in the protective niche and, consequently, their survival (Cavenagh et al., 1993; Barbier et al., 2020).

Another key component that is deregulated in this leukemic microenvironment is angiogenesis. It is well established that, although angiogenesis is considered a physiological phenomenon, it is an essential factor for the viability and development of solid tumors (Falcon et al., 2016). Because of this, the angiogenic phenomenon was initially underestimated in liquid tumors such as AML, which does not have a compact structure. Nevertheless, the formation of vessels is, in fact, crucial for, not only the progression of AML but also for its establishment in extra medullary sites. A parameter for the evaluation of vascularization in leukemic patients is the microvascular density of the bone marrow (MVD). Clinical data revealed that the bone marrow biopsy of AML patients compared to healthy donors has an increased number of sinusoidal blood vessels (Padró et al., 2000). Thus, the degree of MVD might be used as a prognostic marker, making it possible to identify the risk of recurrence and estimate the overall survival of AML patients (Kuzu et al., 2004). Important signaling pathways such as VEGF/VEGFR and Notch/Dll4 (Delta-like ligand 4) are implicated in tumor angiogenesis. Zhang et al. (2013) revealed that untreated AML patients had higher levels of VEGF, VEGFR2, Notch1, and Delta-like ligand 4 (Dll4) compared to healthy donors. Another observation was that the activation of Notch/Dll4 pathway is associated with a poor prognosis. Also, an *in vitro* experiment pointed to a rise in LSC-mediated endothelial cell proliferation that was related to activation of Notch/Dll4 signaling, which led to an increase in metalloproteinase levels, enhancing endothelial cells mobilization and formation of new blood vessels (Zhang et al., 2013). The impact of the interaction between LSCs and endothelial cells on proliferation is mutual. Fiedler et al. showed through a culture of endothelial cells in the presence of the pro-angiogenic factor VEGF led to a dose-dependent increase in granulocyte-macrophage colony-stimulating (GM-CSF), secreted by endothelial cells and known as a mitogen for AML cells (Fiedler et al., 1997).

### The Physiological Reticular Niche

**FIGURE 3:**
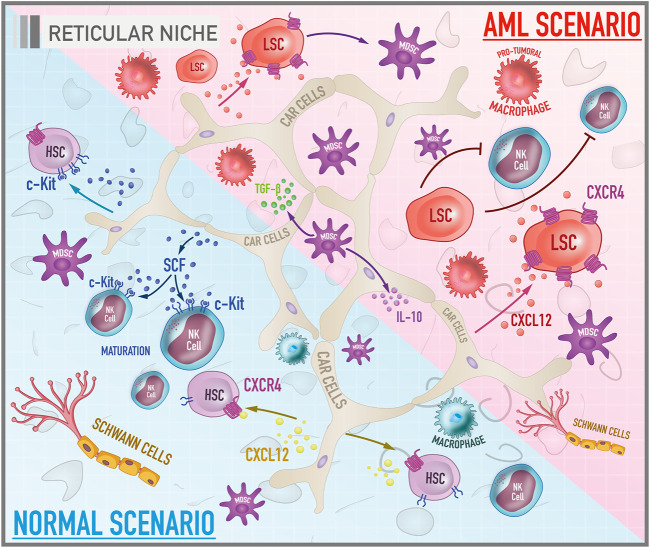
Cellular and molecular components of bone marrow reticular niche in both healthy and leukemic scenarios. The illustration shows the reticular niche which is characterized by being a transitional niche between the endosteal and the vascular ones, responsible for the survival, homing and maintenance of HSCs and other hematopoietic progenitors in a proliferative, but undifferentiated profile. Under physiological conditions, the most important pathways are CXCL-12/CXCR-4 and SCF/c-Kit. In the AML scenario, the CXCL-12/CXCR-4 signaling plays a role in the trafficking and infiltration of leukemic cells into the protective niches of the bone marrow.

Although it is frequently described as containing only two niches (endosteal and vascular), the literature also elucidates the emerging role of a third microenvironment in the bone marrow: the reticular niche. However, there is little documented information about this one. This environment is associated with the maintenance of HSCs in a proliferative profile, similar to the vascular, but in an undifferentiated state, analogous to the endosteal, configuring itself as a transitional niche (Figure 3). Mesenchymal stromal cells, which include CXCL12-abundant reticular (CAR) cells and Nestin-expressing cells, seem to make up the predominant cell group set in this microenvironment (Nagasawa et al., 2011). These cells constitute the dominant stromal cells in the medullary cavity and are located adjacent to the sinusoidal endothelial cells (Weiss 1976; Sugiyama et al., 2006). A study that investigated the location of HSCs and potential cell niches in association with these cells revealed that most HSCs are in contact with CAR cells, responsible for the production of the most essential hematopoietic cytokines such as CXCL12 and SCF (Sugiyama et al., 2006). Not only HSCs, but precursors of B lymphocytes, plasma cells, plasmacytoid dendritic cells, and NK cells also establish interactions with the components of the reticular niche, suggesting that CAR cells may also function as a niche for immune cells (Tokoyoda et al., 2004; Kohara et al., 2007; Noda et al., 2011). Nestin-expressing cells, in turn, are associated geographically with adrenergic nerve fibers and are known to express genes related to the maintenance of HSCs. Depletion of Nestin^+^ MSCs was related to a significant reduction in HSCs pool in the bone marrow and homing of HSPCs (Méndez-Ferrer et al., 2020).

CAR cells are mesenchymal progenitors with the potential to differentiate into adipocytes and osteoblasts for their ability to express adipogenic and osteogenic genes such as PPARγ, runx2, and osterix (Osx). In addition, these cells preserve the proliferation of HSCs and lymphoid progenitors. An experiment involving selective ablation of CAR cells by the administration of diphtheria toxin in an *in vivo* model revealed that the conditioned deletion of these cells results in a decrease in the number, size, and dormancy potentiation of HSCs, as well as an increased expression of genes related to differentiation in a myeloid lineage (Omatsu et al., 2010). Another effect described was the general reduction in CXCL12 and SCF levels. Although other cell groups also produce these cytokines, the study revealed that CAR cells are primarily responsible for supplying these molecules. The potential for differentiation in adipogenic and osteogenic lines was also compromised, as well as for proliferation of erythroid and B cell progenitors, dependent on the support provided by the adipo-osteogenic progenitors (Omatsu et al., 2010). Together, these observations reflect the impact of CAR cells in promoting bone marrow homeostasis.

Consistently with the other described niches, the reticular niche also has important molecule pairs that make possible the maintenance of HSCs in a proliferative, but undifferentiated state. The CXCL12/CXCR4 axis is an example. CXCL12, also known as a factor derived from stromal cells (SDF-1), is a chemokine with chemotactic properties, whose physiological receptor is CXC-chemokine 4 (CXCR4), also known as cluster of differentiation 184 (CD184), a protein with a structure formed by seven transmembrane domains coupled to a heterotrimeric G protein (Baggiolini et al., 1997). The signaling provided by this axis results not only in homing of HSCs, but also promotes the development of the immune system cells such as B lymphocytes (Tokoyoda et al., 2004; Nagasawa 2006). Another important axis of the reticular niche is the SCF/c-Kit axis. Stem cell factor (SCF) is a cytokine that can be soluble or transmembrane. Its c-kit receptor, also known as the cluster of differentiation 117 (CD117), is a tyrosine kinase receptor expressed on the surface of HSCs and hematopoietic progenitors. The binding of SCF to its respective receptor causes c-kit to homodimerize and autophosphorylate in tyrosine residues, triggering a signaling cascade, being able to activate pathways such as RAS/ERK, PI3-kinase, and JAK/STAT (Rönnstrand 2004). The secretion of SCF has similar effects to CXCL-12 such as increased survival, homing, and maintenance of HSCs and other hematopoietic progenitors (Kent et al., 2008).

### The Leukemic Reticular Niche

Among the various soluble factors secreted by microenvironment cells that regulate AML cells, the chemokine CXCL12 is one of the most important. Mesenchymal stromal cells, in particular CAR cells, along with having a notable capacity to differentiate into other cell groups, secrete high levels of CXCL12, making them an interesting and potential therapeutic target in the AML scenario (Figure 3). In physiological conditions, this chemokine is involved in the induction and regulation of trafficking in leukocytes by chemotaxis. However, the interaction of CXCL12 with its CXCR4 receptor appears to be implicated as a critical mediator of the association between stromal and leukemic cells (Peled and Tavor 2013). A study by Möhle et al. showed that leukemic CD34^+^ blasts from AML patients have considerable amounts of functionally active CXCR4 on their surface what may play a role in regulating the trafficking of malignant cells (Möhle et al., 1998). Moreover, it appears that the CXCL12/CXCR4 axis constitutes a critical signaling pathway for the infiltration of leukemic cells into the protective niches of the bone marrow which, under normal conditions, are restricted to hematopoietic cells (Wang and Zhong 2018). Therefore, it is essential to understand the factors that lead to expression of the CXCL12 receptor by leukemic blasts. It is well established that the mutation in the FTL3 gene promotes the activation of CXCR4 signaling in AML cells (Zeng et al., 2009). Another factor that induces the expression of this receptor is the stress induced by chemotherapy. A study by Spoo et al. showed that low expression of CXCR4 in cells of AML patients correlated with a longer relapse-free and longer overall survival compared to patients with intermediate and high levels. Thus, the expression of CXCR4 might be related to the migratory and adhesive behaviour of leukemic cells among the three BM niches, and could be considered as a prognostic predictor for AML patients (Spoo et al., 2007).

## Other Cells Involved in Healthy and Leukemic Bone Marrow.

There are also non-permanent resident cells on the bone marrow microenvironment that migrate and interact with the niches in healthy conditions or during pathological processes such as tumors. They are also reported in and associated with some aspects of physiopathology or immune response to AML.

### Natural Killer Cells

Natural killer cells (NK) are a type of cytotoxic lymphocytes that exhibit an innate and adaptive immune response against tumor cells, including leukemic cells. The bone marrow is the main site of generation and maturation of NK cells in adulthood. NK cells are formed from NK cell progenitors (NKPs) that originate from Common Lymphoid Progenitors (CLPs) based on their interactions with stromal cells, cytokines, growth factors, and other soluble molecules that form a microenvironment characterized by presence of SCF, FLT3L, and IL-7 (Vacca et al., 2011; Di Vito, Mikulak, and Mavilio 2019).

Many strategies used by AML cells to escape the immune system response have been identified, amongst them, the increased expression of inhibitory molecules on the membrane and the secretion of immunosuppressive cytokines, thus creating an immunosuppressive microenvironment that avoids recognition mediated by NK cells, triggering tumor immune escape (Barrett and Le Blanc 2010; Baragaño Raneros et al., 2019).

This tumor immunosuppressive microenvironment results in decreased activity of NK cells by several mechanisms including the defective NK maturation, lysis inhibition by immune checkpoints (PD-1, TIM3, and TIGIT expressed on the cell surface of NK cells, recognize their ligands, which are expressed on the cell surface of AML cells, and as a consequence, activating pathways involved in NK cell regulation are inhibited, promoting NK cell anergy) and modulation of the NK receptor repertoire (NK functions are exhaustively regulated by the balance between activating and inhibitory receptors, however, during AML development the repertoire of NK cells is modified, reducing the level of expression of activating receptors and increasing that of inhibitory receptors) (Chiossone et al., 2017; Baragaño Raneros et al., 2019).

The literature does not make it clear whether NK inhabit the BM and interact with the niches, but possibly the NK migration and interactions are not restricted to one specific site. The ability to perform immune surveillance is conditioned by the expression of a repertoire of inhibitory/activator receptors, chemokine receptors and adhesion molecules that work together to drive NK migration. In a multiple myeloma study, authors demonstrated that CXCR3-and CXCR4 are important receptors to guarantee NK infiltration into bone marrow, and the absence of this signaling is sufficient to decrease NK infiltrating cells. These data are important to sustain the hypothesis of the presence of NK in endosteal, vascular and reticular niches since all of them offer such chemoattractant signals, but also to suggest that any disturbance in the BM microenvironment promoted by AML scenario is able to difficult NK immune surveillance (Raulet, Vance, and McMahon 2001; Morris and Ley 2004; Ponzetta et al., 2015).

### Myeloid-Derived Suppressor Cells

Myeloid-Derived Suppressor Cells (MDSC) are a heterogeneous population of myeloid cells in the early stages of development with an immunosuppressive profile that negatively regulates immune responses and collaborates to tumor development. The literature describes three MDSC subpopulations: the neutrophilic (PMN-MDSC), the monocytic (M-MDSC), and the immature (i-MDSC) (Talmadge and Gabrilovich 2013; Solito et al., 2014; Bronte et al., 2016; Wei et al., 2016; Ibáñez-Vea et al., 2018). These three rare populations can be present in bone marrow, peripheral blood, lymph nodes, spleen, and tumors. However, an altered hematopoietic differentiation pathway leads to an exacerbated production, expansion, and accumulation of these cells as a consequence of pathological conditions such as chronic inflammation, infections, stress, and cancer (Solito et al., 2014; Damuzzo et al., 2015; Bronte et al., 2016; Heine et al., 2017).

In general, MDSC population can suppress specific antitumor adaptive immune responses, increase the growth of already established tumors through citokines secretion (such as IL-10, TGF-β, IL-6, and IL-1β), and provide a spark for the onset of oncogenesis in the inflammatory microenvironment (Kusmartsev and Gabrilovich 2006; Wahl et al., 2006; Gabrilovich and Nagaraj 2009; Ugel et al., 2015; Yaseen et al., 2020; Weber et al., 2021).

Clinical studies have already shown that MDSC is accumulated in the peripheral blood and BM of AML patients comparing to healthy donors (Pyzer et al., 2017). Furthermore, the AML scenario promotes the expansion of the immunosuppressive population of MDSC in the BM (Taghiloo and Asgarian-Omran 2021) and there is a relevant positive correlation between increased MDSC population and poorer prognosis of patients. In fact, the detection of minimal residual disease is directly associated with the presence of MDSC population (Sun et al., 2015).

Although MDSC are not reported occupying a specific site of BM, they are potentially distributed through the three niches following and responding to the blasts presence, and offering back pro-leukemic support.

### M1 and M2 Macrophages

There are resident and monocyte-derived macrophages in the BM, however there is no consensus concerning the specifically niche localization. They have been described to have the ability to adapt to the environment and play roles in maintaining homeostasis and responding to inflammation and infection. Under normal conditions, macrophages have the main function of responding to pathogens and modulating the adaptive immune response through the processing and presentation of antigens. More specifically, M1 macrophages (classically activated), are known to promote Th1 responses, secrete high levels of pro-inflammatory cytokines such as IL-1-beta, TNF-alpha, IL-12, IL-18, and IL-23, they are important in defense against bacterial infections, in addition to presenting high production of reactive oxygen and nitrogen species and causing tissue damage. On the other hand, M2 macrophages (alternatively activated) are described as immunoregulatory and promotors of tissue remodeling. They secrete large amounts of IL-10 and low levels of IL-12 and also secrete CCL17, CCL22, and CCL24 (Gentek et al., 2014; Varol et al., 2015; Wynn and Vannella 2016; Kaur et al., 2017; Kloc et al., 2019; Seyfried et al., 2020).

In cancers, monocytes are recruited by neoplastic and altered stromal cells and differentiate to tumor-associated macrophages (TAM). TAM is activated by an abnormal malignant microenvironment which contributes to tumor progression through promoting genetic instability, nurturing cancer stem cells, supporting metastasis, and regulating adaptive immunity. Depending on the factors offered by the microenvironment, TAM can acquire an M1 (anti-tumorigenic) or M2 (pro-tumorigenic) phenotype. It has been described that TAM preferentially acquires an M2 phenotype, which favors tumor growth, promotes cell survival, proliferation, dissemination, and metastasis (Mantovani et al., 1992; Mantovani et al., 2002; Solinas et al., 2009; Galdiero et al., 2013; Galdiero et al., 2013; Haas and Obenauf 2019; Li et al., 2020).

In leukemia, these cells are currently leukemia-associated macrophages (LAM) (Li et al., 2020). Although the mechanisms are not yet elucidated, Al-Matary et al. (2016) suggest that the LAM protect the AML cells from apoptosis induced by chemotherapy treatment with cytarabine (Al-Matary et al., 2016; Li et al., 2020). Moreover, Yang et al. (2018) observed that bone marrow LAM differentiated with M1 characteristics, while splenic LAM evolved mostly with M2 in AML models (Yang et al., 2018; Li et al., 2020).

### Adipocytes

In the bone marrow, adipocytes constitute a cell group derived from MSC. Although many authors describe these cells close to the endosteal surface, these cells can occupy the entire interior of the medullary cavity, covering up to 70% of the bone marrow in human adults. Nevertheless, the number of these cells may vary according to nutritional conditions and cytotoxic stress. Further studies are needed to confirm the precise location of these cells in the bone marrow and their behavior under physiological and also AML context (Rosen et al., 2009; Reagan and Rosen 2016; Méndez-Ferrer et al., 2020).

In contrast to the idea of being a trivial stock of fat, adipocytes act as regulators of medullary homeostasis by the secretion of molecules such as adipokines (Horowitz et al., 2017). Although it has been described in the literature that chemotherapy can induce adipogenesis with consequences for the bioavailability of drugs in the medullary cavity, the role of adipocytes in leukemogenesis remains controversial (Sheng et al., 2016). Some studies have been done to elucidate the importance of adipocytes to AML development. The research line of Boyd’s group revealed that myelo-erythropoiesis is interrupted in acute myeloid leukemia by the disruption of the adipogenesis (Boyd et al., 2017). In the same way, Battula et al. (2017) showed interruption of the differentiation of mesenchymal cells in the adipogenic lineage in the AML (Battula et al., 2017). Alternatively, Shafat’s studies provide evidences in the opposite direction: adipocytes support AML blasts *in vitro* and *in vivo* by transference of fatty acids to the malignant cells, interfering with metabolism and increasing survival and proliferation. The study also showed that co-culture of adipocytes with leukemic blasts increases the fatty acid-binding protein-4 (FABP4) messenger RNA levels. Interestingly, FABP4 blockage reversed the protection of AML cells mediated by adipocytes (Shafat et al., 2017). Accordingly, Tabe and colleagues raised evidence that the AML cells-adipocytes interactions reduces apoptosis of the monocytic cells by increasing fatty acid β-oxidation (FAO) and expression of genes such as PPARγ, FABP4, CD36, and BCL2. Consequently, the pharmacological inhibition of β-oxidation of fatty acids led to the apoptosis of AML cells. Such observation suggests that the disruption of the oxidation of these energetic molecules can act as a therapeutic strategy in controlling AML progress (Tabe et al., 2017). The association between FAO and leukemic cells was also investigated by Lee et al. (2015), who identified avocatin B, a FAO inhibitor, as a compound with cytotoxic activity in AML cells (Lee et al., 2015). An even more recent study compared adipogenic potential of MSCs derived from healthy donors or AML patients. AML-MSCs presented improved ability to support the survival of leukemia progenitor cells through a mechanism dependent on decreased expression of SOX9 (Azadniv et al., 2020). Taken together, literature give to BM resident adipocytes an important role in normal and AML scenario, what confirm them as interesting cellular targets for new therapies.

### Sympathetic Neural Cells

Although sympathetic neural cells are poorly described in the literature compared to other cells in the bone marrow microenvironment, these cells act on the regulation of the HSCs compartment and, indirectly, is implicated in the AML modulation. A study by Spiegel et al. (2007) revealed that CD34^+^ cells express beta-2 adrenergic and dopamine receptors and their mobility and proliferation can be regulated by adrenaline and noradrenaline (Spiegel et al., 2007). Additionally, unmyelinated Schwann cells seem to express molecules that activate the latent form of TGF-β, which is produced by a diversity of cells, impacting the maintenance and repopulation capacity of HSCs. Consequently, autonomic nerve denervation leads to glial cell death, inducing rapid loss of HSCs (Yamazaki et al., 2011). In myeloproliferative neoplasms, the Schwann cells and Nestin^+^ MSCs are reduced due to neural damage of the bone marrow mediated by IL-1β, produced by malignant cells, which resulted in an expansion of deffective mesenchymal stem and progenitor cells. Pharmacological treatment with β3-adrenergic agonists reduced Nestin^+^ MSCs loss and disrupted myeloproliferative neoplasm evolution (Arranz et al., 2014). Thus, sympathetic neural cells clearly work regulating MSCs response, but there is no robust evidence to confirm consequences for AML development.

## The Extramedullary Acute Myeloid Leukemia Microenvironment

Extramedullary acute myeloid leukemia (eAML) is defined as a tumor infiltration composed of myeloid blasts outside bone marrow, what includes both hematopoietic (spleen, liver) and non-hematopoietic tissues (skin, gums, and central nervous system) (Comings et al., 1965; Byrd et al., 1995; Shallis et al., 2019). eAML may occur simultaneously with or before bone marrow presentation, and also during relapses (with and without prior allogeneic stem cell transplant) (Solh et al., 2016). Around 0.8–2% of AML cases will develop extramedullary manifestation (Movassaghian et al., 2015; Goyal et al., 2017). Most cases are related to *de novo* AML but can also appear in acute blastic transformation of myelodysplastic syndrome, myelodysplastic/myeloproliferative neoplasms or myeloproliferative disorders (Traweek et al., 1993). Clinical presentation in the skin or gums is often concomitant to bone marrow involvement with isolated sites in lymph nodes, intestine, mediastinum and orbit (Neiman et al., 1981). The three most common sites of presentation are connective/soft tissues (31.3%), skin/breast (12.3%), and digestive system (10.3%) (Goyal et al., 2017). The prognostic impact of extramedullary disease in AML is widely discussed in literature and some authors defend that extramedullary disease brings an independent prognostic effect, others describe as inferior outcomes (Shimizu et al., 2013).

The tissue architecture of eAML lesions is simpler comparing to the bone marrow niches already described in this review. The leukemic infiltrate is histologically characterized by hyperleukocytosis with a monotone accumulation of myeloblasts/monoblasts that interact closely with stromal cells (Goyal et al., 2017; Shallis et al., 2021).

There is extent information regarding how mesenchymal stromal cells regulate AML inside bone marrow, what includes cytokine/chemokine secretion, microRNA-containing exosomes release and cell-cell contact by gap junctions, for example (Barrera-Ramirez et al., 2017; Forte et al., 2020; Kouzi et al., 2020). Because their presence in a variety of non-hematopoietic tissues in the body, they potentially would exert an additional role in extramedullary infiltration (Carter et al., 2016), but such mechanisms was not explored in the literature.

Although it has been reported an angiogenic process during eAML lesions constitution (Piccaluga et al., 2018), it remains unclear if AML blast cells establish some functional crosstalk with endothelial cells similarly to the phenomena observed in vascular niches in the bone marrow.

Chemotaxis and cellular adhesion seems to be critical for the extramedullary infiltration. Notwithstanding the equivalent expression of CD56 in leukemic cells from patients with or without eAML, some evidences support that CD56, a glycoprotein responsible for cell-cell adhesion, could promote the attachment of leukemic blasts to adipose, skeletal muscle, gastrointestinal, testicular, and brain tissue. In addition, although there was not confirmed evidence of a cause-effect relationship, it is clarified that overexpression of CD11b (β2-integrin member macrophage-1 antigen) in myelomonocytic and monocytic blasts positively correlates to eAML episodes (Ganzel et al., 2016; Shallis et al., 2021). Further contribution have been provided by studies involving skin eAML biopsies from pediatric patients that presented overexpression of CXCR4 and CXCR7, which are bone marrow specific homing chemokine receptors and whose connection with skin CXCL12 may result in the evolution of skin eAML (Faaij et al., 2010).

A fibrotic pattern with collagen deposition is also reported in the eAML sites (Cunningham et al., 2019). In other cancer models and tissues, fibrosis is associated to the frequency and activation of MDSC and M2 macrophages (Hammerich and Tacke 2015; Tang et al., 2019). A study by Hui Sun and colleagues showed that MDSC levels positively correlated with extramedullary infiltration in *de novo* AML patients (Sun et al., 2015). If eAML present similar mechanisms, the axis MDSC-M2 should be considered in participating on the extramedullary infiltration. Nonetheless, the role of M2 macrophages has not yet been elucidated in the context of eAML.

The current effort to investigate AML niches as targets for therapy certainly will naturally include extramedullary lesions in the focus of the researches, and relevant discoveries may give to eAML additional prognostic value.

## Perspectives for Acute Myeloid Leukemia Therapies Targeting Bone Marrow Niches.

The poor outcomes achieved by the current AML therapies are constantly encouraging researchers to propose new and better strategies to mitigate the leukemia development and evolution. The increased understanding about BM niches and about how these microenvironments cross-talk and regulate AML has allowed the discovery of potential novel approaches. Interestingly, part of these studies is testing well known drugs which act on common pathways and receptors shared by niches and blasts. Other groups of trials are targeting some niche-specific molecules. Table 1 summarizes some of the main ongoing clinical trials related to BM-associated molecules described in this review.

**TABLE 1:** Studies registered on ClinicalTrials.gov investigating bone marrow niches-related molecular targets in AML.

ClinicalTrials.gov Identifier	Stage	Intervention	Condition or disease	Age (Years)
ENDOSTEAL NICHE				
NCT04460963	Phase 1	Adrenomedullin inhibition	AML	18 +
NCT00827138	Phase 1	New inhibitor of BCR-ABL, Flt3, Tie2 and other kinases	CML, AML	18 +
NCT01555268	Phase 1	Neutralizing peptibodie agains Angiopoietin 1/2	AML	18 +
VASCULAR NICHE				
NCT00542971	Phase 1/2	Co-treatment with VEGFR-kinase inhibitor	MDS, AML	15–60
NCT00071006	Phase 2	VEGFR-, PDGFR- and BCR-ABL-kinase inhibitor	MDS, AML	18 +
NCT00015951	Phase 2	Co-treatment with monoclonal antibody against VEGFR	Leukemias	18–120
NCT04518345	Phase 1/2	Novel specific kinase-inhibitor against ALX receptors	AML	18 +
NCT03616470	Phase 3	Specific E-selectin antagonist	AML	18–75
RETICULAR NICHE				
NCT00989261	Phase 2	FLT3 inhibitor	AML	18–85
NCT02634827	Phase 2	Pan kinase-inhibitor	AML	60 +
NCT02984995	Phase 2	FLT3-inhibitor	AML	20 +
NCT00045942	Phase 1/2	PKC-inhibitor	MDS, AML	18 +
NCT01445080	Phase 1/2	VEGFR-, PDGFR-kinase inhibitor	AML, solid tumor	2–21
NCT02954653	Phase 1	Monoclonal antibody against CXCR4	AML	18 +
NCT01546038	Phase 2	Sonic Hedgehog-inhibitor	MDS, AML	18 +

CML, Chronic Myeloid Leukemia; MDS, Myelodisplastic Syndrome.

Membrane receptors such as VEGFR, PDGFR, c-Kit, and CXCR4 are important targets in these studies not only due to their relevance in the maintenance of BM homeostasis and niches dynamics, but also because there are several inhibitors and antagonist already tested in other models and patients. Most of them presented successful results in solid or other hematological neoplasms, and the current understanding about vascular and endosteal contributed to the interesting in investigating them on AML.

Other groups of niches-related molecules have been also explored. The blockage of ligands, such as angiopoietin and CXCL12, or the inactivation of adhesion molecules, such as E-Selectin, have been tested with neutralizing peptides or antagonist small molecules. New combination protocols with traditional chemotherapy plus niche-related drugs are also common attempts observed in the ongoing trials.

The hypothesis of all these protocols is the same: disturbing the AML bone marrow organization by interfering with niche-related mechanisms and damaging proliferation, differentiation, or resistance to apoptosis. Besides additional basic studies, hematologists wait for further phase-3 clinical trials to finally confirm the efficacy of this tendency for new protocols.
